# Co-producing rapid research: Strengths and challenges from a lived experience perspective

**DOI:** 10.3389/fsoc.2023.996585

**Published:** 2023-03-23

**Authors:** Karen Machin, Prisha Shah, Vicky Nicholls, Tamar Jeynes, TK, Kylee Trevillion, Norha Vera San Juan

**Affiliations:** ^1^NIHR Mental Health Policy Research Unit Lived Experience Working Group, Division of Psychiatry, University College London, London, United Kingdom; ^2^NIHR Mental Health Policy Research Unit, Health Service and Population Research Department, King's College London, London, United Kingdom; ^3^Rapid Research Evaluation and Appraisal Lab (RREAL), Department of Targeted Intervention, University College London, London, United Kingdom

**Keywords:** co-production, Lived Experience Researchers, rapid research, meaningful involvement, experts by experience

## Abstract

The Lived Experience Researchers (LERs) of the Mental Health Policy Research Unit (MHPRU) reflect on the experience of conducting rapid co-produced research, particularly during the first year of the COVID-19 pandemic. Throughout this perspective article, we introduce requirements for co-production applying the 4Pi Framework, reflect on specific characteristics of co-production in rapid research, discuss strengths and challenges for involvement of LERs in rapid research, and lastly provide recommendations to achieve meaningful involvement. Incorporating meaningful co-production is an augmentation to any research project, with several benefits to the research, to the team, and to individual researchers. Particularly in the case of rapid research, that aims for efficient translation of knowledge into practice, involvement of experts by experience will be key. The work conducted by the MHPRU LERs presented in this paper demonstrates the viability, value, and potential of this way of working.

## 1. Introduction

The Lived Experience Working Group (LEWG) of people with personal experience of mental health issues and involvement in research, has been part of the Mental Health Policy Research Unit (MHPRU) almost since it was established at University College London (UCL) and King's College London (KCL) in 2017 (more information here https://www.ucl.ac.uk/psychiatry/service-user-and-carer-involvement-mhpru).

When the LEWG was recruited, attention was paid to recruiting as widely as possible in terms of ethnicity, age, geography, gender and mental health service experiences. Involvement of people with lived experience is a central part of the Unit and, within this, co-production activities have been undertaken and members of the LEWG reflect on these in this article.

We discuss co-production of mental health research, including the impact on researchers and research outcomes. We use the “4Pi framework” (Faulkner et al., 2014) with its five elements of involvement: Principles, Purpose, Presence, Process, and Impact, underpinned by a 5th “P” of Power. We then place this in the context of rapid research in relation to our work conducted during the COVID-19 pandemic. To our knowledge, co-production within rapid research is not a field that has been explored before, rapid research understood as efficiently and collaboratively conducting research for an applied purpose. We argue for the value of Lived Experience Researchers' (LERs') active involvement in rapid research study teams and conclude with recommendations for good practice and further research.

There are multiple definitions and interpretations of co-production and involvement, and how these are enacted in practice, with the two terms often used interchangeably. One definition is:

“[…] an approach in which researchers, practitioners, and the public work together, sharing power and responsibility from the start to the end of the project, including the generation of knowledge” (NIHR Involve, 2019, p. 4).

Terms used within the context of involvement include Lived Experience Research, Patient and Public Involvement (PPI), and Service User Involvement. It is important to note the distinctions between being a “participant,” and actively being involved in research (Colder Carras et al., 2022).

### 1.1. COVID-19 research

When the pandemic began, everyone had to respond quickly, and many academics turned to rapid research, although many did not name it as such at that point. MHPRU brought together a team of Lived Experience Researchers (LERs), including existing LEWG members, to conduct research interviews, participate in analysis, and co-author publications. An example of this was an interview study of mental health service users' early experiences of the COVID pandemic within which the team of LERs interviewed 49 people with pre-existing mental health conditions and supported the rapid analysis and writing of four papers (Gillard et al., 2021; Johnson et al., 2021; Sheridan Rains et al., 2021; Shah et al., 2022). This article reflects on experiences of research involvement, including in these studies, from the perspective of researchers with lived experience of mental health challenges or distress, as either service users or carers.

## 2. Requirements for co-production

“When patients are involved in research, this will enhance the societal impact and relevance” (Groot et al., 2022, p. 1).

Good guidance on the planning and design of involvement in research has been established for nearly 20 years (Faulkner, 2004). Additionally, there are various frameworks to guide the implementation of involvement, participation, and co-production of research, each with a different emphasis. In a systematic review of 65 frameworks, Greenhalgh et al. (2019) suggest five categories of: power-focused; priority-setting; study-focused; report-focused; and partnership-focused. One framework developed by people with lived experience is the 4Pi framework co-produced by the National Survivor User Network (NSUN) (Faulkner et al., 2014) and originally established to support co-production in services (NSUN, 2018). Researchers highlight that this framework has universal relevance and is firmly grounded in service user experience and partnership working (Matthews et al., 2019). It has previously been used in a project to evaluate involvement in research (The contribution of the voluntary sector to mental health crisis care in England, n.d.). Consequently, we use it here as a framework to discuss the requirements for co-production.

4Pi stands for Principles, Purpose, Presence, Process, and Impact. Within a rapid project, it may feel more important to jump to the **Process** of involvement: to address questions such as *how can we do it* and *what steps do we need to take*. But the initial elements ensure that co-production starts from a base which values lived experience. Consideration of **Principles** offers an opportunity for a research team to reflect together on their values and fundamental reasons for co-production. **Purpose** requires defining an objective or aim for involvement, which can be evaluated later for **Impact. Presence** asks the team to question who is involved to ensure the inclusion of people with a range of experiences relevant to the specific project and with attention to groups who may otherwise be excluded and unheard.

**Impact** in co-produced research is frequently overlooked or considered as an after-thought rapid research. In an evaluation of 15 Patient and Public Involvement (PPI) strategy documents using the 4Pi Involvement Standards, only two met all the criteria for assessing impact (Matthews et al., 2019). Although some mentioned impact, very few gave consideration to mechanisms facilitating measurement or to the context of their intended purpose or desired outcome. Many tools for measuring impact exist, but they are not used reliably or consistently (MacGregor, 2021). We recognize some of the challenges in measuring impact and suggest caution that it does not become a tick-box exercise.

Underpinning any co-production and involvement is also the issue of **Power**, which is emphasized in all decisions taken within a project. One of the hallmarks of high-quality co-production is *equal involvement at every stage and every level*. Too often, LERs are brought in after initial decisions are taken, and are consequently unable to contribute to defining the optimal research question. Similarly, with short, sessional work, LERs can easily be omitted from major decisions or elements within a project.

LERs are often not fully immersed in a team. While this brings the advantage of additional objective, and, independent perspectives, it is essential not to overlook the potential impact on individuals of being an outsider. LERs commonly follow career and life paths which differ from regular academics. Their **Presence** brings a rich diversity of perspective to the work. In addition to the lived experience relevant to the research topic, we introduce different ways of working. We value opportunities for collaborating and learning, especially the provision of peer reflective spaces which allow us to share our personal responses to the work and create a culture of care and mutual respect. The opportunity to add “lived experience commentaries” to MHPRU papers has been valued by all (e.g., Barnett et al., 2021; Schlief et al., 2022), and, we suggest, should be standard practice to ground the research and enhance the understanding in a real-life context.

People with the decision-making power for a project need to have the skills and experience to understand the landscape of lived experience research, including an awareness of involvement frameworks, and they need to understand the impact of their decisions on LERs as individuals and professionals within a team. Communication skills are at the heart of this, alongside reflections around different working tools and how they might feel to people without institutional access to technology platforms and software. A routine task like receiving emails can feel burdensome when a person is only meant to be involved for a defined number of hours a month, as is often the case for LERs. The team needs to be very clear about expectations and time commitments.

Academic researchers can benefit from professional development opportunities to help them recognize the advantages of working with lived experience colleagues and the benefits that co-production brings to projects. The inherent reflexivity around power and relationship also has a positive effect upon team culture and staff wellbeing support and should be considered an investment in the organization as well as good practice, and an enhancement to the research at hand.

## 3. Co-production in rapid research

Rapid research places specific and additional pressures on co-production in research: we highlight the factor of time which has an impact on resources. Ensuring an adequate budget for co-production and involvement costs at the early stages is crucial. The context of the pandemic generated a high demand for research providing new opportunities for involvement and co-production. Remote working facilitated involvement while simultaneously demanding LERs develop new skills and build experiences within their research “portfolios” (another example of impact). For some people, working from home felt more inclusive, including making use of transferable skills and working strategies which may previously have been seen as limitations requiring adjustments. Remote working overall facilitated working nationally which involved regularly attending meetings, carrying out data collection and collaborative writing, although the resources and equipment provided by LER were often assumed. A LEWG member described “*My laptop is no longer fit for purpose to keep up with the shift to primarily working over Zoom and using high-spec software compared to exchanging occasional emails before Covid-19.”*

Responding to the 4Pi factors requires a team to reflect together, having time to think about each step and be inclusive about the different standpoints of team members to reach agreements, stepping back to rethink established processes. We find it helps to be realistic at the start about the boundaries and constraints of the project and which elements can or cannot be co-produced. In the context of rapid research, it's even more essential to build in mechanisms for evaluating impact and outcomes from the beginning, and to consider this from multiple perspectives: impact on the research, impact on lived experience researchers, and impact of lived experience input into broader end outcomes. For example, in the common task of choosing illustrative phrases for qualitative reporting there can be a fine line between memorable and triggering that requires room for team reflexivity.

The impact of involvement on LERs has itself been the subject of research. Faulkner and Thompson (2021) explore the “emotional labour” experienced by user researchers in mental health research, describing the negotiation of identity, the emotional work of using and embodying lived experience, and aspects of the working environment. These descriptions resonate with experiences from the academic team, particularly during the intense period of COVID-19. While our expertise has a beneficial impact on the direction, processes and interpretation of the research, being routinely exposed to potentially emotionally distressing material can intersect with personal experiences of mental health and being from minoritised groups. However, discussing “emotional labour” can highlight the tensions around perceived fragility or acknowledged expertise, with its echoes of “skivers” and “strivers” (Carr, 2019).

The input from LERs needs greater recognition and responsibility within powerful, influential and multidisciplinary academic structures to ensure people are adequately supported emotionally and practically. Following a round of rapid research in the early stages of the pandemic, we co-developed and completed a survey to evaluate our experiences and gauged further support and training needs. The MHPRU team responded to our requests for additional support structures by developing a system of regular weekly peer reflection sessions. Our access to these peer-facilitated spaces enabled mutual support, listening, understanding and kindness. Access also to a monthly academic researcher-facilitated space provided some level of supervision and an opportunity to raise current issues that could be addressed by the team. Outside of formal working structures, LERs began to get to know each other, perhaps in a more accelerated way, and form stronger support bonds.

A final example of impact were the positive experiences of members of the MHPRU academic team:

“*Working with LER colleagues has had a hugely positive impact on my practice. With each collaborative piece of work we do, my knowledge and insights develop in ways that wouldn't be possible without lived experience involvement. Our collaborations have also helped me establish more innovative research practices and to generate research knowledge that is richer and more novel. My LER colleagues continue to teach me new things, which is a fundamental part of research practice.” Kylee Trevillion, Deputy Director of MHPRU, King's College London*.

## 4. Discussion

Through team reflective discussions we identified strengths and challenges for meaningful involvement of LERs in rapid research. Time was a key factor in all challenges, shared across research teams although perhaps felt more acutely by LERs who sometimes described feeling external to a team. Responding quickly to the pandemic disruption, with the rapid adaptation to working online, was a challenging time for many researchers. Having systems in place for communications so that people are clear about their roles and to ensure that actions are taken in a timely manner, is crucial for meaningful inclusion of LERs.

Other challenges are common to any co-production process but may be more obvious where work needs to be completed quickly. Power dynamics and assumptions around LERs' abilities and capacity can be barriers to equality which is a core value of co-production (Carr, 2019), creating increased pressures during intense periods of work. Team members have different skills and experiences of lived experience research as well as personal, individual experiences of distress and feelings about disclosure. Co-production requires time to understand the variety of personal perspectives and potentially arrange for individual training needs to be addressed within the timescales for academic researchers as well as LERs.

It takes time to build the relationships of trust and equality required for successful co-production. The MHPRU team had a head start when required to respond rapidly to the COVID restrictions: the LEWG team were already in place and familiar to the academic team. Longer-term partnerships are needed to ensure that the benefits of lived experience research can be maximized. Research funders need to place emphasis on building the capacity of both research teams and lived experience researchers to ensure successful co-production and lived experience leadership (Jones et al., 2021).

Greater reflection on the limitations of rapid research on coproduction is necessary, as true co-production has always been a slow process. However, this would necessitate an entire chapter in itself to expand on topics such as how the lack of time and resources has the potential to lead to involvement feeling tokenistic, especially where a team of LERs is not already in place. The necessarily slow pace of building mutual and trusting relationships is at the foundation of good team working, but can conflict with the requirements for rapid results. Such challenges can be particularly noticeable where a team brings together a range of different experiences and perspectives, both lived and learned, and including different demographic characteristics and experiences of distress.

We emphasize that meaningful involvement has the potential to offer important benefits to a research project where time and resources allow. A team of LERs can root the study in a breadth of experiences as survivor activists, facilitators, transformers, and humanisers (Daya et al., 2020) contributing and creating debate and discussion which adds to the knowledge of the whole team. LERs ensure that time and resources spent on a project are well spent, studies are relevant, and results will have impact. Our own team was intersectionally diverse, including people from a range of different ethnic backgrounds who helped in areas such as identifying gaps in research design and in recruiting from more diverse communities than is typical. The 4Pi process encourages the research team to pay attention to **Presence** to ensure that relevant people are included. Additionally attention to **Impact** mitigates against tokenistic involvement.

Dissemination as part of the **Impact** can be overlooked as an integral part of the research process and lost as academic teams move onto their next project. Where dissemination is seen as an activity that occurs after the completion of a project, LERs may be unintentionally excluded, exaggerating the emotional labor of coproduction. However, such exclusion is a missed opportunity for the study: LERs will have a range of additional networks as well as skills, which may provide additional benefits for ensuring the results of a study reach a wider audience beyond that reached by traditional academics.

LERs are often at a disadvantage to evaluate the level of co-production because they do not know what they do not know. Unequal power dynamics may mean that they are not privy to discussions around budgets and decisions that impact on levels of involvement. Effective co-production will only ever be achieved by organizations sharing their power–we feel this can only benefit the quality, diversity, outcomes, and impact of rapid research.

### 4.1. Recommendations

Our recommendations for involvement in rapid research are firmly based on the principles for co-production of any research. However, co-production involves the use of reflexive thinking which requires time and is counterintuitive to rapid research. Teams therefore need to develop methods that allow for this to be efficiently carried out (e.g., Collaborative Matrix Analysis conducted in Vera San Juan et al., 2021).

Our first recommendation concerns who is involved. Building long term relationships between LERs and academic teams establishes trust and working practices before they are needed for rapid research. Relationships can also be built with a range of LERS to ensure **diverse** experiences are included and encouraged, with newer recruitment building capacity alongside the development of leadership opportunities. It is also important and ethical to embed an approach of reaching out to communities and activists who may have an interest in research that is being conducted.

A second recommendation is about ensuring time for communication and reflection, both for the academic team as a whole and for the LERs as a peer group. Reflective spaces are often overlooked but are particularly valued by LERs. Communications need to be timely and accessible, in a variety of agreed formats, both within the team and wider dissemination of research results. Reflective spaces that have worked for us include meeting up beforehand to check our backgrounds and reflective methods we have used. This leads to agreeing a purpose for the reflective space, focusing on the experience/feelings/emotional labor of the work, rather than on deeper issues, which might not be possible to deal with in that setting. The space is to be used as people need in terms of being able to speak about both positive and negative experiences. Others can respond as they feel happy, and a facilitator has a very light touch, moving things forward, giving all a chance to speak, and reminding everyone of ground rules.

We recommend the practice of LE commentaries within published papers, where the most important reflections materialize and are shared with readers.

**Thirdly**, the impact of co-production and involvement needs to be recorded and evaluated to build evidence, and we recommend use of the 4Pi framework. Mechanisms for feedback need to be included alongside a process to implement change where relevant.

Our **fourth** point concerns the resources for involvement and coproduction. Where a team of LERs has been established, such costs are more easily estimated. Without an existing team, costs for items such as reflective spaces and technical equipment can be overlooked. Such resources need to be considered in funding proposals and funders need to be aware of such expectations.

**Finally**, a reminder that meaningful co-production is an ongoing process that should precede the initiation of the project and continue until dissemination. People with lived experience often hear the regrets of researchers when good ideas are suggested but it is too late to act on them. LERs should be involved in shaping the whole research agenda as well as defining the research question from an early stage through to dissemination, including sharing the impact of the involvement itself.

Building on these fundamental recommendations, we would like to suggest development of participatory research to include research topics and questions which are led by LERs. However, a first step must be to build capacity within academic teams for LER leadership.

The perspectives and learnings of academic researchers on lived experience involvement is perhaps under-researched, and LERs could lead co-production to build this evidence. As a small first step, mirroring the lived experience commentaries of academic papers within the PRU, as a team of LER authors we have invited an Academic Commentary for this paper from our academic PRU colleagues not working through a lived experience lens (see Box 1).

Box 1 **Academic experience commentary written by Sonia Johnson, Bryn Lloyd-Ev-ans, and Alan Simpson on 17/07/2022.**

We were fortunate that established relationships with our LER group allowed us to set up and conduct the MHPRU interview study rapidly and collaboratively at the onset of the COVID pandemic. We could also draw on existing experiential, theoretical, and methodological knowledge from colleagues, including LERs, in conducting participatory, coproduced qualitative analysis. We agree that building long-term relationships between LERs and academic teams is hugely helpful.

We can now see that we underestimated the emotional effects on LERs of this project re-searching impacts that they too were experiencing. We are glad of the constructive suggestions made by LER colleagues about developing support systems, like the reflective space group. We will be better prepared in future projects, and now incorporate such systems as standard practice. The necessity of switching to online working also brought sustainable benefits for collaborative working. It overcomes problems of geography and logistics, and allows meetings to be arranged at short notice, or LERs to dip in and out of meetings or switch cameras off as required, should meetings become stressful. Remote working continues to be at the wM of our working practices.

Papers from this project had a clear focus on exploring inequalities and which groups were most affected by the pandemic. This reflects the values and lens of our LER colleagues and is an example of how they enriched the project.

Doing research together-interviewing and analyzing data, writing collaboratively -breaks down barriers beyond what advisory groups can achieve. It helps us to see our the LERs with whom we work primarily as colleagues. Working so collaboratively in a large group during the early months of the pandemic and lockdown met needs for many of us to connect with others and to feel we were contributing something of value.

### 4.2. Conclusion

In conclusion, we feel that investment in meaningful co-production is an augmentation to any research project, with several benefits to the research, to the team, and to individual researchers. Within rapid research, the key challenge is time, chiefly the time to build the working relationships at the heart of co-production. However, the work of the MHPRU LERs in responding to the requirements for rapid research during the pandemic, demonstrated the viability, value and potential of this way of working.

“*Rapid research went against all of my instincts in terms of time for reflection and discussion. However, we somehow built that in. We blazed through it and it was published swiftly enough to be of use in improving service design - we had also managed to make researchers think about different approaches. It isn't perfect, but it is an example of steps in the right direction, which will hopefully make a difference to future research projects and teams” [LEWG member]*.

## Data availability statement

The original contributions presented in the study are included in the article/supplementary material, further inquiries can be directed to the corresponding author.

## Author contributions

KM, PS, VN, TJ, TK, KT, and NV contributed to the conception, discussions, and initial drafting of the manuscript. KM, PS, and NV completed the final version and formatting. All authors contributed to the article and approved the submitted version.
